# The effect of calcium gluconate administration during cardiopulmonary bypass on hemodynamic variables in infants undergoing open-heart surgery

**DOI:** 10.1186/s43044-022-00266-w

**Published:** 2022-04-13

**Authors:** Seyedeh Zahra Faritous, Saeed Rajabzade Zaree, Zohreh Morshedizad, Amir Hossein Jalali, Soha Mehrabi Mahani, Maziar Gholampour

**Affiliations:** Rajaie Cardiovascular Medical and Research Center, Iran University of Medical Sciences, Vali_e_Asr St., Tehran, Iran

**Keywords:** Calcium gluconate, Cardiopulmonary bypass, Hemodynamics, Open-heart surgery

## Abstract

**Background:**

The incidence of complications after heart surgery is a critical factor in disability, deaths, lengthening hospital stays, and increasing treatment costs. The metabolic balance of certain hormones and electrolytes is necessary for proper cardiac function. In children, various biochemical conditions may cause calcium depletion during heart surgery. The purpose of this study was to determine the effect of calcium gluconate administration during cardiopulmonary bypass on hemodynamic variables and clinical outcomes in infants undergoing open-heart surgery. This study was conducted at Rajaie Cardiovascular Medical and Research Center in 2021 using a controlled randomized clinical trial. A total of 60 patients with open-heart surgery weighing up to 10 kg were included in the study. The first group received an intravenous injection of calcium gluconate 20 min after opening the aortic clamp, and the second group was monitored as a control group. Data collection tools included checklists containing demographics, surgical information, and intensive care unit measures.

**Results:**

The Chi-square test or Fisher's exact test showed that the frequency distribution of gender, blood group, Rhesus factor (RH), and clinical diagnosis in the two groups of intervention and control was not statistically significant (*p* < 0.05). The mean and standard deviation of Ejection Fraction (EF) changes (before and after) were 13.27 ± 9.16 in the intervention group and 8.31 ± 9.80 in the control group (*p* = 0.065). The results of two-way repeated measures ANOVA showed that mean systolic blood pressure (*p* = 0.030), mean diastolic blood pressure (*p* = 0.021), mean heart rate (*p* = 0.025), mean arterial pressure (*p* = 0.020), mean pH (*p* < 0.001), and mean hemoglobin (*p* = 0.018) in the intervention, and control groups were statistically significant.

**Conclusions:**

In the present study, unlike systolic pressure, mean diastolic blood pressure decreased, and mean arterial pressure increased significantly. As a result, the slope of changes during the study period was different in the intervention and control groups.

## Background

Certain hormones and electrolytes must be in balance for healthy cardiac function [1, 2]. Cardiopulmonary bypass (CPB) disrupts electrolyte and hormone metabolism [3–5] and the transition from CPB to spontaneous heart function is limited to just a few minutes [6]. CPB termination requires specific cardiac contractors, such as inotropes, that improve cardiac function [7–9]. The selection of inotropic and vasopressor drugs depends on the patient's cardio pathology and hemodynamic state [6, 10]. The use of beta-adrenergic mimetics such as epinephrine and calcium is common during patient isolation and resumption of Ejection Fraction (EF) [9]. As an essential component of the maintenance and regulation of normal heart function, calcium plays a critical role in cardiomyocyte contraction and expansion [6, 11–13]. Calcium-sensitive contractile proteins and stored calcium can alter the contractile force of the heart. In previous cases, modulating calcium homeostasis through inotropic drugs caused increased calcium contractions in myocardial cells [14].

Calcium plays a crucial role in cell contractions and relaxation, with positive inotropic and chronotropic effects. Calcium levels may decrease during CPB due to a difference in biochemical conditions of acid and base as well as changes in water and electrolytes [6, 15–17]. Among the causes of hypocalcemia are hemodilution from prime crystalloid fluid, calcium band with citrate in the blood, calcium band with heparin injected during prime, and calcium band with albumin given to increase plasma oncotic pressure. The effects of hypercalcemia, particularly in severe cases, include decreased myocardial function and vasodilation [18]. Myocardial infarction causes decreased cardiac contraction, resulting in the use of calcium as a treatment in cardiac surgery [14]. These reasons lead anesthesiologists to use calcium chloride to correct myocardial contractions during CPB separation [6, 19].

Low extracellular calcium concentrations are common following pediatric open-heart surgery [20]. This is because children have limited intracellular calcium reservoirs, making them more dependent on extracellular calcium. Due to the likely association between serum ionized calcium levels and complications as well as the hemodynamic status during and after surgery, this study investigated the impact of calcium gluconate on hemodynamic variables and its clinical consequences, during and after cardiopulmonary bypass.

## Methods

### Objective

This study aimed to determine the effect that calcium gluconate administration during cardiopulmonary bypass has on hemodynamic variables in infants who have undergone open-heart surgery at Shaheed Rajaie Cardiovascular, Medical & Research Center in Tehran.

### Trial design

**Fig. 1:**
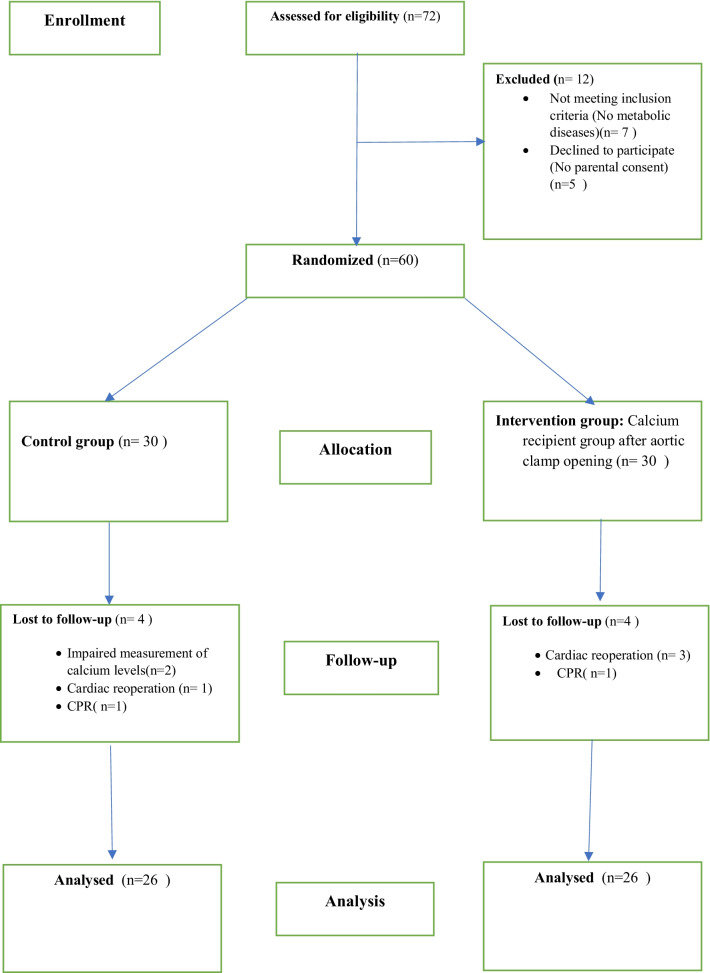
CONSORT diagram: selection, evaluation, and follow-up of the participants

The study was performed in 2021 on children at Shaheed Rajaie Cardiovascular, Medical & Research Center as a randomized controlled trial with a single blinding control in which the intervention was hidden from the participants. Neither the patient nor the perfusionist was aware of the contents of the injected solution (whether or not it contained calcium). The study population included all infants with congenital heart disorders (Table 1) who were candidates for open-heart surgery and weighed one to ten kilograms. We reported this trial and described the interventions following the CONSORT guidelines and checklist. To randomize the patients, the computer program [21] created two groups of 30 patients, in the form of block randomization of size 6 (including 10 blocks of 6). Since the study was conducted during the peak period of COVID-19 in Iran, this limitation may have affected the total number of surgeries performed (Fig. 1).

**Table 1:** Primary cardiac diagnosis for 72 patients

Primary cardiac diagnosis	Number of patients with diagnosis
Ventricular septal defect	20
Tetralogy of Fallot	16
Double outlet right ventricle	14
Atrioventricular septal defect	12
Atrial septal defect	10

### Inclusion and exclusion criteria

Inclusion criteria include parental consent to take part in the research project and infant patients undergoing open-heart surgery weighing up to 10 kg, normal and lower ionized calcium during cardiopulmonary bypass, absence of any metabolic and systemic diseases that cause changes in calcium gluconate levels, no preoperative digoxin use, no hypophosphatemia, no reoperation, or emergency. We excluded patients with impaired measurement of serum calcium gluconate for any reason (including failure to send a sample, laboratory errors in measurement, measurement problems, sending an insufficient sample for measurement, etc.), cardiomyopathy, and any resuscitation operation during surgery.

### Sample size

Following a study by Johnston, Robertie, and their colleagues in 1992 in the United States [9], the following equations determined the sample size needed:

1 $$n_{1} = \frac{{\left( {Z_{{1 - \frac{\alpha }{2}}} + Z_{1 - \beta } } \right)^{2} \times \left( {\sigma_{1}^{2} + \frac{{\sigma_{2}^{2} }}{k}} \right)}}{{\Delta^{2} }},\quad n_{2} = k \times n_{1}$$

2 $$\alpha = 0.05 \to Z_{{1 - \frac{\alpha }{2}}} = 1.96$$

3 $$\beta = 0.20 \to Z_{1 - \beta } = 0.85$$

4 $$\sigma_{1} = 3\,{\text{mmhg}}$$

Therefore, the required sample size is 26 for both the calcium gluconate group and the control group. Based on the drop of 15% in patients after exclusion criteria, we needed 30 patients in each group and 60 infants with congenital heart disease undergoing open-heart surgery.

### Methods

A nasal cannula with a flow rate of 2 lit/min was used to provide oxygen to patients as they entered the operating room. Before starting anesthesia, patients were hooked up to pulse oximetry monitors and brain oxygen saturation instruments. Anesthesia was induced using Midazolam (0.6 mg/kg), Fentanyl (1–3 µg/kg), and Rocuronium (0.6 mg/kg) after complete monitoring (EKG, Pulse Oximetry, NIBP), followed by TIVA (Midazolam (2–8 µg/kg/min), Fentanyl (0.7–1 µg/kg/h), and Atracurium (0.005–0.01 mg/kg/min). As part of the evaluation, an arterial line (invasive blood pressure) was set up, NIRS (cerebral oximetry) was performed, the central temperature was monitored through the use of an esophageal temperature monitor, and venous access (femoral or subclavian or internal jugular) was performed. The induction process was started with 3–4% Sevoflurane in the absence of peripheral blood vessels. The patient was mechanically ventilated with an anesthesia machine before receiving a central venous catheter. The maintenance dose of 1% isoflurane or sevoflurane was inhaled until the cardiac bypass was performed and then the intravenous infusion was performed through the central venous catheter.

All patients underwent cardiopulmonary bypass (Stockert5), and BICAVAL cannulation was used in all cases. A ringer lactate solution and albumin are used to prime the cardiopulmonary circuit. The body temperature of the patients was gradually cooled to reach the desired surgical temperature to perform the surgery properly. During the bypass procedure, heparin was administered at 300 units/kg to prevent the formation of blood clots. If needed, extra heparin was used during the procedure to avoid the Activated Clotting Time (ACT) dropping below 400.

Another point to note is that the cardioplegia solution was the same in both groups (control and intervention). During myocardial protection, an initial dose of 30 cc/kg of cold blood cardioplegia was injected and repeated every 20 min, and 15 cc/kg of warm blood cardioplegia was injected before aortic declamping.

The study group received 100 mg/kg intravenous calcium gluconate at a rate of 25 mg/kg/min, up to 1 g of calcium gluconate 20 min after opening the aortic clamp, while the control group was evaluated as a control.

The hemofiltration technique uses the convection method to remove water and other low-weight molecules from plasma under a hydrostatic pressure gradient. The hemodilution effects of CPB are more evident in children because of the larger priming volume, and hemofiltration is well known for its beneficial effects on children. We rinsed a spiraflo polysulphone ultrafilter with 1000 ml of saline and inserted it between the arterial tubing and the cardiotomy reservoir. In order to reach a cardiotomy reservoir level of zero after rewarming, hemofiltration was performed at an adjusted rate. The hemofiltration rate was adjusted in order to achieve a zero level of cardiotomy reservoir after rewarming. During the rewarming process, the CBP flow did not change [22, 23].

In this study, PaCO_2_ alpha-acetate was used, and brain oxygenation was monitored continuously. We maintained hematocrits over 24% in all groups. If needed, blood transfusions were administered. During pumping, mean arterial blood pressure (MAP) was measured using a pressure transducer mounted at the heart's level and linked to a monitor. Throughout the study, sensors were placed in the nasopharynx of the patients to continuously monitor their body temperatures.

Upon admission to the ICU, the patient underwent complete monitoring (EKG, Pulse Oximetry, NIBP). The ventilation settings were adjusted according to the patient's condition. For example, early extubation was considered in Fontan and Glenn surgery. If a patient is at risk for pulmonary hypertension, inhaled Nitric Oxide (iNO) and high FiO_2_ may be required. Ventilator settings for a biventricular child are as follows: FiO_2_: 40% or more to achieve target saturation, PEEP:5 cmH_2_O, Tidal Volume: 6–8 cc/kg, PIP: 12–15 cmH_2_O, RR: Normal for age (to achieve PCO_2_ targets).

CXR was also performed in the ICU to determine the correct position of the tracheal tube, lung fields, lung perfusion, and the position of drains. Also, it was determined that the acceptable amount of bleeding in the first hour of the operation was 2–4 cc/kg, and in the second and third hours, 2 cc/kg or less. The constant monitoring of temperature in the ICU was also an important aspect, especially for patients with low cardiac output. Also, electrolytes (sodium, potassium, and magnesium) are continuously monitored in the ICU. It is recommended that the level of ionized calcium be higher than 1 mmol/L (in infants, it should be above 1.2 mmol/L). Calcium should be administered intravenously based on the amount of elemental calcium administered. A starting dose of 10–20 mg/kg of elemental calcium is recommended.

### Research variables

The study included two groups of patients. Approximately 20 min after opening the aortic clamp, the first group received a dose of 100 mg/kg calcium gluconate per minute up to a maximum of 1 g, and the second group was evaluated as a control group [24]. The following hemodynamic variables were measured: systolic and diastolic blood pressure (SBP, DBP), heart rate (HR), and mean arterial blood pressure (MAP). Following the opening of the aortic clamp and the completion of cardiopulmonary bypass, calcium injections were administered to the patient at the beginning of the Intensive Care Unit (ICU) stay and also two, four, six, and eight hours later.

### Data analysis

A statistical analysis of the collected data was performed using SPSS software version 22. A two-sample independent t-test was used to compare the means of quantitative variables between the two groups. The normality of the frequency distribution of quantitative variables was evaluated by the non-parametric Kolmogorov–Smirnov test, and no violation of this assumption was observed (*p* < 0.05). Also, the Chi-square test or Fisher's exact test was used to compare the frequency of qualitative variables between the two groups. To compare the mean of quantitative variables "before induction of anesthesia, after induction of anesthesia, pump completion, at the entrance to the ICU, 2, 4, 6, and 8 h after entering the ICU", in the two groups, Two-way repeated-measures ANOVA was used, and "group effect, time effect, group interaction, and time" were evaluated. Homogeneity of covariance matrices was evaluated by Box’s M test, and no violation of this assumption was observed (*p* < 0.05). The significance level in the tests was considered 0.05.

### Ethical considerations

The ethical considerations of this research are as follows:

1. Obtaining the permission of the ethics committee of the Shahid Rajaei Cardiovascular Training, Research and Treatment Center
2. Obtaining written informed consent from sick parents of children under 7-years-old, to participate in the study
3. Assuring all participants' families that their information will be kept confidential
4. Giving the patient's family the right to choose to enter or withdraw from the study
5. Follow-up and treatment of any accident or complication attributed to the research without imposing a cost on the patient
6. Providing the research results to the interested centers and research units if they wish
7. Registration of the trial on www.IRCT.ir with registration code IR.RHC.REC.1400.042

## Results

The Chi-square and Fisher's Exact test showed that the frequency distribution of gender, blood group, RH, and clinical diagnosis in the two groups of intervention and control were not statistically significant (*p* > 0.05). Also, the *t*-test of two independent samples showed that the mean height of patients in the intervention and control groups was not statistically significant (*p* = 0.108), while the mean of age, weight, and Body Surface Area (BSA) in the control group were significantly more than the intervention group (*p* < 0.05). Calcium is injected based on weight, therefore, heterogeneity of weight, age, and BSA will not affect the comparison of hemodynamic variables and clinical outcomes between groups (Table 2).

**Table 2:** Descriptive statistics of demographic variables

	Group	N	Mean	SD	SE mean	*p* value
*Continuous variables*						
Age (month)	Calcium infusion	26	11.01	10.10	1.98	0.028*
	Control	26	17.38	10.22	2.00	
Weight (kg)	Calcium infusion	26	6.63	2.61	.51	0.039*
	Control	26	8.01	2.04	.40	
Height (cm)	Calcium infusion	26	68.3	12.54	2.46	0.108
	Control	26	73.8	11.88	2.33	
Body surface area (m^2^)	Calcium infusion	26	.352	.10	.019	0.048*
	Control	26	.404	.08	.016	
Duration of aortic cross-clamp (min)	Calcium infusion	26	55.73	6.37	3.23	0.105
	Control	26	54.11	5.71	2.7	
Bypass time (min)	Calcium infusion	26	145.8	10.92	4.09	0.113
	Control	26	146.96	10.01	3.85	
RBC transfusion (mL/kg)	Calcium infusion	26	8.19	2	0.52	0.072
	Control	26	8	1.89	0.42	

Chi-square tests	Statistics	*p* value
*Categorical variables*		
Blood group (A, B, AB, O)	.589	.951
RH (positive, negative)	.991	.350
Diagnosis (cyanotic, non-cyanotic)	.391	.755
Gender (male, female)	.392	.531

*p-value < 0.05

We compared the amount of blood received by both groups (during and following bypass), and there were no significant differences. Furthermore, there was no significant difference in the mean duration of bypass in the intervention and control groups. Similar findings were found for the average duration of cross-clamping.

According to the results, 26 patients in the intervention group received inotropes as compared to 24 in the control group (92.3%). The Fisher's exact test determined that the rate of inotropic use in both intervention and control groups was not statistically significant (*p* = 0.4990). In addition, the results of the study revealed that in the intervention group 5 patients (19.2%) after surgery and 21 patients (80.8%) received inotropes during and after surgery, while in the control group one patient (4.2%) during surgery, 6 (25%) after surgery, and 17 (70.8%) received inotropes during and after surgery. Based on Fisher's exact test, the time of receiving inotropes for the intervention and control groups was not significantly different (*p* = 0.610).

The results also showed that the mean and standard deviation of Ejection Fraction (EF) before surgery was 54.62 ± 5.08% in the intervention group and 52.54 ± 5.79% in the control group (*p* = 0.175). After surgery, the mean and standard deviation of the EF were 41.35 and 7.29% in the intervention group and 44.23 and 7.03% in the control group (*p* = 0.135). Additionally, the mean and standard deviation of EF changes (before and after) for the intervention group were 13.27 and 9.16%, respectively, whereas for the control group were 8.31 and 9.80 (*p* = 0.065).

The effect of the group (between groups) was statistically significant (*p* = 0.030) according to two-way repeated measures analysis (ANOVA). As a result, the mean systolic blood pressures in the intervention and control groups are statistically significantly different (Table 3). As a result, the intervention group's overall mean systolic blood pressure was lower than the control group during the study period. The above analysis (Table 3) also showed an effect of time (intragroup effect) (*p* < 0.001). At different times studied, the mean SBP of the two groups (in a total of all studied groups) is statistically significant. In other words, the mean systolic blood pressure in both groups decreased significantly during the study period. There was no significant interaction between group and time (*p* = 0.057). As a consequence, both the intervention and control groups experienced similar changes in mean systolic blood pressure during the study period. Therefore, the calcium gluconate intervention did not have a significant effect on systolic blood pressure.

**Table 3:** Statistics of systolic blood pressure

	Group	Mean	SD
SBP (before anesthesia)	Calcium infusion	93.00	11.713
	Control	96.54	9.030
	Total	94.77	10.508
SBP (after anesthesia)	Calcium infusion	97.23	8.135
	Control	96.73	8.938
	Total	96.98	8.466
SBP (after finishing the pump)	Calcium infusion	88.46	14.749
	Control	93.46	12.710
	Total	90.96	13.863
SBP (upon entering the ICU)	Calcium infusion	85.04	21.195
	Control	93.54	21.418
	Total	89.29	21.529
SBP (2 h after entering the ICU)	Calcium infusion	92.50	13.796
	Control	98.00	18.042
	Total	95.25	16.143
SBP (4 h after entering the ICU)	Calcium infusion	84.04	14.309
	Control	97.31	21.412
	Total	90.67	19.235
SBP (6 h after entering the ICU)	Calcium infusion	84.12	16.729
	Control	94.23	17.303
	Total	89.17	17.608
SBP (8 h after entering the ICU)	Calcium infusion	83.19	16.783
	Control	93.96	14.256
	Total	88.58	16.348

	*F* statistics	*df*	*p* value
*Repeated ANOVA results*			
Group effect	4.993	1	.030
Time effect	4.122	7	.001
Group and time effect	1.978	7	.057

Table 4 shows that the results of the two-way analysis of variance with repeated measures indicated that the effect of group (between groups) was statistically significant (*p* = 0.021). Therefore, there is a statistically significant difference in diastolic blood pressure between the intervention and control groups over the total study period. In other words, the overall mean diastolic blood pressure level during the study period was significantly lower in the intervention group than in the control group. Mean diastolic blood pressure in the two groups differed significantly at different times during the study, with a significant decrease during the study (*p* = 0.011). The interaction between group and time was also significant (*p* = 0.002). The intervention group's slope of change in mean diastolic blood pressure is different from that of the control group. Calcium injection lowered diastolic pressure in the intervention group, but according to routine care in the ICU, the diastolic pressure climbed, then decreased, until the end of the study. Although in the control group, diastolic pressure continued to increase up to 2 h after entering the intensive care unit and then gradually decreased until the end of the study.

**Table 4:** Statistics of diastolic blood pressure

	Group	Mean	SD
DBP (before anesthesia)	Calcium infusion	54.62	8.357
	Control	56.35	8.314
	Total	55.48	8.300
DBP (after anesthesia)	Calcium infusion	55.50	8.724
	Control	53.35	6.125
	Total	54.42	7.542
DBP (after finishing the pump)	Calcium infusion	51.35	10.914
	Control	51.54	8.918
	Total	51.44	9.869
DBP (upon entering the ICU)	Calcium infusion	49.73	13.809
	Control	54.46	13.447
	Total	52.10	13.705
DBP (2 h after entering the ICU)	Calcium infusion	54.04	9.115
	Control	58.69	11.513
	Total	56.37	10.546
DBP (4 h after entering the ICU)	Calcium infusion	48.35	7.552
	Control	57.42	11.254
	Total	52.88	10.538
DBP (6 h after entering the ICU)	Calcium infusion	48.27	9.159
	Control	56.73	10.857
	Total	52.50	10.824
DBP (8 h after entering the ICU)	Calcium infusion	47.88	9.626
	Control	55.58	8.981
	Total	51.73	10.002

	*F* statistics	*df*	*p* value
*Repeated ANOVA results*			
Group effect	5.692	1	.021
Time effect	2.657	7	.011
Group and time effect	3.302	7	.002

The heart rate variable showed statistical significance between the groups (*p* = 0.025), which means that each group had a significantly different heart rate. During the study period, the mean heart rate of participants in the intervention group was significantly higher than that of those in the control group. The effects of time (intragroup effects) are also statistically significant in Table 5 (*p* < 0.001). This indicates that the mean heart rate was statistically significant in all groups studied at various times. The heart rate of both groups increased significantly during the period of study. Furthermore, the statistical analysis also showed that there was no significant interaction between time and the group (*p* = 0.468). Thus, the slope of change in mean heart rate during the study period was similar between the two groups. Therefore, the calcium gluconate intervention had no significant effect on heart rate.

**Table 5:** Statistics of diastolic heart rate

	Group	Mean	SD
HR (before anesthesia)	Calcium infusion	131.65	21.352
	Control	117.12	18.771
	Total	124.38	21.215
HR (after anesthesia)	Calcium infusion	124.54	18.940
	Control	120.00	18.760
	Total	122.27	18.805
HR (after finishing the pump)	Calcium infusion	137.50	14.160
	Control	127.92	18.661
	Total	132.71	17.099
HR (upon entering the ICU)	Calcium infusion	147.12	22.850
	Control	134.50	18.602
	Total	140.81	21.590
HR (2 h after entering the ICU)	Calcium infusion	148.58	22.622
	Control	136.77	21.049
	Total	142.67	22.441
HR (4 h after entering the ICU)	Calcium infusion	146.62	22.427
	Control	139.77	17.562
	Total	143.19	20.241
HR (6 h after entering the ICU)	Calcium infusion	142.54	22.288
	Control	138.46	17.952
	Total	140.50	20.142
HR (8 h after entering the ICU)	Calcium infusion	141.42	21.034
	Control	137.77	17.303
	Total	139.60	19.158

	*F* statistics	*df*	*p* value
*Repeated ANOVA results*			
Group effect	5.337	1	.025
Time effect	14.553	7	.001
Group and time effect	.950	7	.468

The mean arterial pressure (MAP) was the last variable examined in this study. The two-way analysis of variance with repeated measures revealed that the effect of group (between groups) was statistically significant (*p* = 0.020). Thus, MAP in the intervention and control groups are statistically significant over the time studied. MAP is significantly lower in the intervention group than in the control group (Table 6). Furthermore, in both groups, the mean arterial pressure increased significantly over time (*p* = 0.002). There is a difference in the slope of changes in the mean of MAP between the intervention and control groups during the study period (*p* = 0.005). Specifically, following calcium gluconate injection, the mean arterial pressure in the intervention group decreased, and then, following routine care in the intensive care unit, the mean arterial pressure increased, then decreased after the study ended. After entering the intensive care unit, mean arterial pressure increased for 2 h before showing a gentle decrease in the control group.

**Table 6:** Statistics of mean arterial pressure

	Group	Mean	SD
MAP (before anesthesia)	Calcium infusion	67.41	8.938
	Control	69.74	7.494
	Total	68.58	8.251
MAP (after anesthesia)	Calcium infusion	69.41	7.480
	Control	67.81	6.221
	Total	68.61	6.859
MAP (after finishing the pump)	Calcium infusion	63.72	11.750
	Control	65.51	9.484
	Total	64.62	10.611
MAP (upon entering the ICU)	Calcium infusion	61.50	15.889
	Control	67.49	15.633
	Total	64.49	15.896
MAP (2 h after entering the ICU)	Calcium infusion	66.86	10.097
	Control	71.79	13.209
	Total	69.33	11.904
MAP (4 h after entering the ICU)	Calcium infusion	60.24	9.329
	Control	70.72	14.174
	Total	65.48	13.004
MAP (6 h after entering the ICU)	Calcium infusion	60.22	11.101
	Control	69.23	12.545
	Total	64.72	12.580
MAP (8 h after entering the ICU)	Calcium infusion	59.65	11.639
	Control	68.37	10.169
	Total	64.01	11.682

	*F* statistics	*df*	*p* value
*Repeated ANOVA results*			
Group effect	5.737	1	.020
Time effect	3.359	7	.002
Group and time effect	2.990	7	.005

## Discussion

The purpose of this study was to determine the effect of calcium gluconate administration during cardiopulmonary bypass on hemodynamic variables in infants undergoing open-heart surgery.

In our study, the rate of inotropic use and the time of inotropic reception for the intervention and control groups were not statistically significant. The study by Johnston et al. in 1992 compared ephedrine, calcium chloride, and placebo in isolating patients from cardiopulmonary bypass. 36 patients aged 43 to 80 years who were candidates for cardiac surgery had their hemodynamic variables assessed. This study found that calcium chloride could not enhance right ventricular function during detachment from cardiopulmonary bypass in patients with moderate hypocalcemia. On the other hand, in patients with normal preoperative ventricular function, ephedrine improved right ventricular function and arterial blood pressure better than placebo or calcium chloride [9]. Moreover, Kimura et al. conducted a retrospective study at Okayama Hospital in Japan in 2017 to determine the relationship between ionized calcium concentrations and outcomes 24 h after heart surgery in children. The results of their study indicated a statistically significant association between the levels of antihypertensive drugs and serum ionized calcium levels [25].

In the study of hemodynamic variables, the overall mean systolic blood pressure during the study period in the intervention group was significantly lower than the control group. Also, in all two groups, mean systolic blood pressure decreased significantly during the study period. However, the slope of changes in mean systolic blood pressure during the study period was similar in the intervention and control groups. In other words, the effect of calcium gluconate intervention on systolic blood pressure was not significant. In the study of diastolic blood pressure, the overall mean during the study period was significantly lower in the intervention group than in the control group. In addition, in all two groups, mean diastolic blood pressure showed a significant decrease during the study period. But unlike systolic pressure, the slope of changes in mean diastolic blood pressure during the study period was different in the intervention and control groups. The overall mean heart rate at study time in the intervention group was significantly higher than the control group. In both groups, the mean heart rate increased significantly during the study period. The slope of changes in mean heart rate during the study period was similar in both intervention and control groups. In other words, the effect of calcium gluconate intervention on heart rate was not significant. In the intervention group, overall mean arterial pressure was lower than in the control group at study time. The mean arterial pressure increased significantly in both groups during the study period, and therefore, the slope of changes in mean arterial pressure during the study period was different in the intervention and control groups. In a similar clinical trial to determine the effect of calcium injection after cardiopulmonary bypass in 20 patients, DeHert et al. found that calcium injection in the early period after CPB improved left ventricular systolic function but temporarily impairs diastolic function [14]. Desai et al. in their study observed the relationship between ionized calcium and arterial pressure in 112 patients admitted to the intensive care unit in three groups: treated with vasopressor drugs, receiving vasodilators, and receiving diuretics. Hypotension and hypocalcemia are associated with clinical significance, they concluded. Additionally, ionized hypocalcemia was common in this patient group and was associated with overt hypotension and the requirement for vasopressor support [26]. Following open-heart surgery, Murdoch et al. injected 12 children with 10% calcium chloride at a dose of 0.1 ml kg for a maximum of 5 ml over 5 min. After injection of 10% calcium chloride, a significant increase in mean systemic arterial blood pressure occurs as a direct result of an increase in systemic vascular resistance index and a decrease in cardiac parameters [27].

Upon cessation of circulation, Shapira et al. evaluated the hemodynamic effects of intravenous calcium chloride injections in 26 patients. Calcium chloride was injected into 18 patients in the first group in a bolus dose of 10 mg/kg. For the second group of eight patients, after injection of calcium chloride bolus with the same dose, its infusion was followed at a rate of 1.5 mg/kg/min for 10 min. In addition to myocardial contractile velocity, aortic blood flow, electrocardiogram, and left ventricular pressure, systemic, pulmonary, and left atrial pressure were continuously measured. Initial hemodynamic improvements following calcium chloride injections included increased myocardial contractility rate, heart rate, mean blood pressure, and stroke volume index. A similar pattern of hemodynamic response was observed in both groups. Approximately one minute after calcium chloride injection, the heart rate returned to control, myocardial contraction rate and mean blood pressure remained high, and heart rate decreased. Systemic vascular resistance increased gradually and increased significantly in the second group at 3 min and in the first group at 6 min. We conclude that after cardiopulmonary bypass, calcium chloride injection causes an immediate and continuous increase in myocardial function and blood pressure and causes a temporary increase in cardiac index with a gradual increase in systemic vascular resistance [5].

Resnick [28] explains two mechanisms through which calcium may affect blood pressure. The depletion of calcium may result in a significant reduction in calcium in all cell membranes, thus decreasing the stability of smooth muscle cell membranes. In addition, when the calcium concentration is optimal, the smooth muscle cells' membranes are stabilized, thereby preventing calcium from entering the cell and preventing vasoconstriction [28, 29]. Calcium, along with other ions such as potassium and magnesium, acts to stabilize cell membranes and maintain ionic balance [30]. Renin-angiotensin (RAS) is another system that controls blood pressure by affecting calcium metabolism and vascular tone [31–33]. A rise in intracellular calcium leads to an increase in vascular smooth muscle tone, peripheral vascular resistance, and the sympathetic response, resulting in an increase in blood pressure [34–36]. Several studies have demonstrated that hypocalcemia can lower blood pressure during weaning from the CPB. Calcium can currently be helpful in weaning off CPB, according to these results [37]. Calcium administration has improved cardiac output and the mean arterial pressure in several studies. A study showed an increase in cardiac index, stroke volume, and mean blood pressure after administering 1.5 mg/kg/min of calcium [5, 38, 39]. The evidence supporting the beneficial effects of calcium (such as increasing the variables mentioned above) is limited in the case of Vasoplegia and moderate contractility reduction [5]. The calcium ions play an essential role in the excitation–contraction coupling process of Cardiomyocytes [40, 41]. As the concentration of intracellular Ca^2+^ increases, the cardiac troponin c conformation is altered, causing the tropomyosin complex to separate and expose the myosin-binding site on the actin molecule, resulting in muscle contraction [41]. There was a biphasic response to CaCl_2_ administration in a study by Shapira [5]. In the first phase, the cardiac index increased, and the SVR decreased. In the second phase, the SVR gradually increased and continued to increase throughout the study. In the first phase, both systolic and diastolic pressure increased immediately. The increase in blood pressure is due to the increase in cardiac index in the first phase and the increase in the SVR in the second phase. Also, the increase in the cardiac index in the second phase is due to the increase in myocardial contractility along with the increase in stroke volume despite the increase in afterload [5]. Ionized calcium is known to affect hemodynamic variables in various ways. This effect can either be direct or will increase the release of catecholamines from the adrenal glands and sympathetic fibers. According to [42], CaCl_2_ administration increased SVR immediately after CPB onset. Other physiological reasons for this reaction include the use of CPB, systemic hypothermia, surgical trauma, and changes in catecholamine levels, which modulate the response to CaCl_2_ administration [5].

## Conclusions

In the present study, according to the initial hypotheses, the average systolic blood pressure and mean heart rate in both intervention and control groups were similar during the study period. Therefore, calcium gluconate intervention did not have an apparent effect on them. No statistically significant difference in inotropic rate, inotropic reception time, and EF was found between the intervention and control groups. Unlike systolic pressure, mean diastolic blood pressure decreased, and mean arterial pressure increased significantly. Therefore, the slope of change during the study period was different between the intervention and control groups. Considering the involvement of other factors, such as magnesium ions and potassium, it seems that additional research in other settings is required to generalize the results of this study.

## Data Availability

Not applicable.

## Abbreviations

RH: Rhesus factor
ANOVA: Analysis of variance
EF: Ejection fraction
CPB: Cardiopulmonary bypass
TIVA: Total intravenous anesthesia
ACT: Activated clotting time
MAP: Arterial blood pressure
SBP: Systolic blood pressure
DBP: Diastolic blood pressure
HR: Heart rate
ICU: Intensive critical unit
BSA: Body surface area
CPR: Cardiopulmonary resuscitation
RBC: Red blood cells
FFP: Fresh frozen plasma
Plt: Platelet
NIRS: Near infra-red spectroscopy
EKG: Electrocardiogram
NIBP: None invasive blood pressure
iNO: Inhaled nitric oxide
PEEP: Positive end-expiratory pressure
PIP: Peak inspiratory pressure
RR: Respiratory rate
CXR: Chest X-ray
RAS: Renin-angiotensin system
